# Study on Water Damage of Asphalt–Aggregate Based on Molecular Dynamics

**DOI:** 10.3390/ma18010209

**Published:** 2025-01-06

**Authors:** Shenghao Wang, Yan Chen, Lihua Wang, Naixin Cui, Chunfeng Li, Shifu Sun

**Affiliations:** 1 College of Civil Engineering and Architecture, Shandong University of Science and Technology, Qingdao 266590, China; 15662036109@163.com (S.W.); c15163365297@163.com (N.C.); m19862206909@163.com (C.L.); ssfsyf@163.com (S.S.); 2 College of Architecture Engineering, Qingdao Binhai University, Qingdao 266555, China; 18754280953@163.com

**Keywords:** emulsified asphalt–aggregate interface, water damage, adhesion work, molecular dynamics

## Abstract

To investigate the water damage at the interface between emulsified asphalt and aggregate under the action of external water infiltration, firstly, cetyltrimethylammonium bromide was used as an emulsifier to prepare emulsified asphalt in the laboratory, and its basic properties were tested. Then, based on molecular dynamics, an emulsified asphalt–aggregate interface model with different water contents was constructed to calculate the adhesion work of the emulsified asphalt–aggregate interface. The results show that the simulated values of emulsified asphalt density, cohesive energy density, and solubility are in good agreement with the experimental values. Under the same water content, the adhesion force between asphalt and three oxides (CaO, Al_2_O_3_, SiO_2_) is arranged in the following order: CaO > Al_2_O_3_ > SiO_2_. The bonding performance of an alkaline aggregate to asphalt is better than that of an acid aggregate. The van der Waals force plays a major role in the adhesion performance of an emulsified asphalt mixture, and electrostatic force plays a secondary role. Under the action of external force, the macroscopic failure mode of the emulsified asphalt–aggregate is as follows: the alkaline oxide-emulsified asphalt system is cohesive failure; the acid and neutral oxide-emulsified asphalt system is adhesive failure; the enrichment of water molecules at the interface is the main factor causing water damage.

## 1. Introduction

In recent years, Chinese road construction has developed rapidly. Asphalt concrete pavement has been increasingly applied to the road construction of various grades due to its characteristics of smooth surface, comfortable driving, wear resistance, environmental protection and noise reduction, short construction period, simple maintenance and repair, and recyclable regeneration. Asphalt roads will have disease problems after a long period of use. To avoid the impact on the normal traffic and driving safety of the road section, it is necessary to detect and repair the disease in time. However, the traditional repair technology has the problems of large engineering quantity, serious pollution, and waste of resources. The emergence and application of emulsified asphalt can solve the above problems to a certain extent. It can make full use of the existing materials and replace the traditional hot mix asphalt with emulsified asphalt that can be produced at room temperature, which can reduce the energy consumption and environmental pollution based on achieving an ideal repair effect [1].

However, emulsified asphalt has the disadvantages of low adhesion, low early strength, poor storage stability, and poor adhesion. Studying the adhesion performance of an emulsified asphalt cold recycled mixture, especially the contact state and adhesion strength formed by the internal interface of an emulsified asphalt mixture under the action of water molecules, is helpful to understand the formation mechanism of the water damage of a mixture, and to carry out targeted prevention and control of a weak interface, reduce the generation of interface damage under the action of water, and prolong the service performance and service life of recycling engineering. It is of great positive significance.

Many scholars have conducted a lot of research on asphalt–aggregate interface water damage and molecular dynamics simulation. Lv Zhitian et al. used the molecular dynamics method to study the influence of the chemical composition and temperature of different aggregates on the adhesion performance of the asphalt–aggregate interface. They believed that the adhesion work of the asphalt–aggregate interface increased first and then decreased with an increase in temperature, it was similar to the cohesion work of asphalt at room temperature, and it was easy adhesion failure and cohesion failure at the same time. In addition, the adhesion work between an alkaline oxide and asphalt was greater than that between an acidic oxide and asphalt [2]. Wu Meng et al. studied the influence of different emulsifier types on the water damage resistance of emulsified asphalt and believed that a cationic emulsifier has better adsorption to aggregate than an anionic emulsifier [3]. Si Youxiang et al. constructed an asphalt–water layer-aggregate model by artificially adding water molecular layers and studied the effect of water on the pull-out strength of asphalt–aggregate. They believed that adding aquifers to the asphalt–aggregate interface model could simulate water damage. With the increase in interfacial water content, the interfacial energy between the asphalt and aggregate decreases, resulting in a weaker adhesion effect [4]. Hu Tao et al. discussed the effect of residual water in foamed asphalt molecules on the adhesion properties of foamed asphalt and aggregates. They believed that the presence of residual water in foamed asphalt reduced the adhesion properties of foamed asphalt, and the adhesion properties decreased with the increase in residual water content [5]. Although many scholars have conducted a lot of research on water damage and molecular dynamics simulation of the asphalt–aggregate interface, these studies pay less attention to the influence of external water migration into asphalt on the interface bonding characteristics. Because of this, based on the molecular dynamics, the emulsified asphalt–aggregate model with different water contents was established by using lammps software, and visualized by using olex2 to explore the influence of internal water molecule migration on the bonding characteristics of emulsified asphalt after immersion.

## 2. Preparation of Emulsified Asphalt

### 2.1. Raw Material

Kunlun-70 # A-grade asphalt was selected for indoor testing, and its main performance indicators are shown in Table 1. The remaining indicators met the requirements of “Standard Test Methods of Bitumen and Bituminous Mixtures for Highway Engineering” (JTG E20-2011 [6]).

The four-component content of asphalt was determined according to the “Test method for separation of asphalt into four fractions” (NB/SH/T 0509-2010 [7]). The test results are shown in Table 2.

The emulsifier used in the test was analytically pure cetyl trimethyl ammonium bromide. The test water was tap water.

### 2.2. Emulsified Asphalt Production Steps

The solid content of emulsified asphalt is 55%, the amount of emulsifier is 1.5% of the total mass of emulsified asphalt [8], and the mass ratio of each raw material of emulsified asphalt is asphalt/water/emulsifier = 550:442:8. The emulsified asphalt production steps are as follows:

After heating the water to 80 °C, add an emulsifier, stir evenly, so as to make the emulsifier solution. Heat the asphalt to 150 °C, so that all the asphalt melts. Open the colloid mill; first pour in an appropriate amount of hot water above 90 °C for preheating for 2 min, empty the hot water, add the emulsifier solution at 80 °C to the colloid mill, adjust the colloid mill speed to 650 rpm, grind for 3 min, then slowly add the molten matrix asphalt to the colloid mill, adjust the colloid mill speed to 1200 rpm, and shear and emulsify for 5 min to obtain cationic emulsified asphalt. After testing, its main performance indicators were as shown in Table 3.

## 3. Modeling and Methods

### 3.1. Emulsifier Model

**Figure 1:**
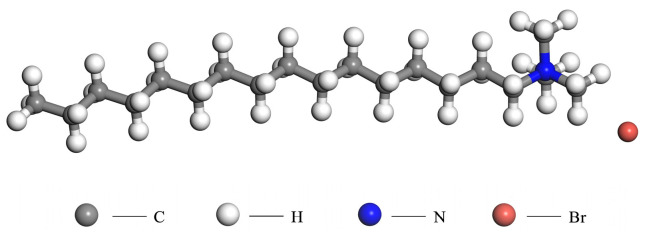
Molecular model of CTAB emulsifier.

In this paper, cetyltrimethylammonium bromide (CTAB, molecular formula C_19_H_42_BrN) was selected as an emulsifier to construct an emulsified asphalt model. As a cationic emulsifier, CTAB has excellent surface activity and biodegradability and has good compatibility with other cationic, non-ionic, and zwitterionic surfactants. It is widely used in asphalt emulsification. The initial molecular configuration was established by using packmol v20.12.0 [9] software and visualized by using olex2 v1.1.02 software. The molecular model of CTAB emulsifier is shown in Figure 1.

### 3.2. Emulsified Asphalt Model

**Figure 2:**
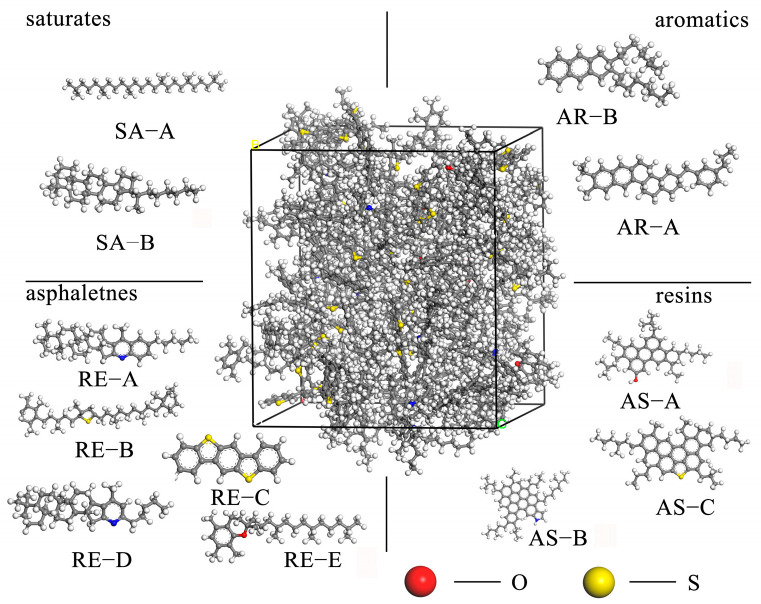
Asphalt model.

At present, the asphalt molecular models commonly used by scholars include the average molecular model, the three-component molecular model, the four-component molecular model (saturates, aromatics, resins, asphaltenes), and the twelve-component molecular model. As shown in Figure 2, the four-component, twelve-molecule model proposed by Li et al. [10] was used to construct the matrix asphalt model. By adjusting the number of water molecules, the molecular structure model of emulsified asphalt with different water contents [5] (water contents of 0.1%, 0.5%, 1.0%, 1.5%) was established. The mass ratio of each component is shown in Table 4.

**Figure 3:**
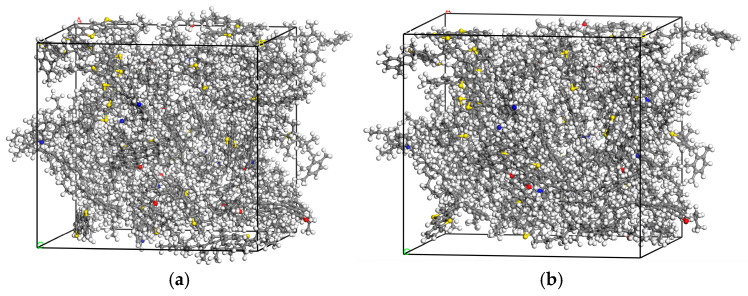
Emulsified asphalt model: (**a**) three-dimensional full-cycle emulsified asphalt model; (**b**) restricted emulsified asphalt model.

The three-dimensional periodic emulsified asphalt model and the restricted emulsified asphalt model with Z-axis direction as the shrinkage boundary condition were established by using lammps Shell 1.2 software to combine each molecule in the Monte Carlo way, as shown in Figure 3.

After the construction of the emulsified asphalt cold recycled mixture was completed, the asphalt emulsion was demulsified due to the evaporation of water molecules. At this time, the emulsified asphalt is only composed of asphalt molecules and emulsifier molecules. The emulsified asphalt residue model as a description of the product after demulsification is more consistent with the description of aggregate adhesion. Therefore, this paper uses this model to represent “emulsified asphalt”. Unless otherwise stated, the emulsified asphalt mentioned in the subsequent part of this paper refers to the residue model after demulsification.

### 3.3. Asphalt–Aggregate Interface Model

**Figure 4:**
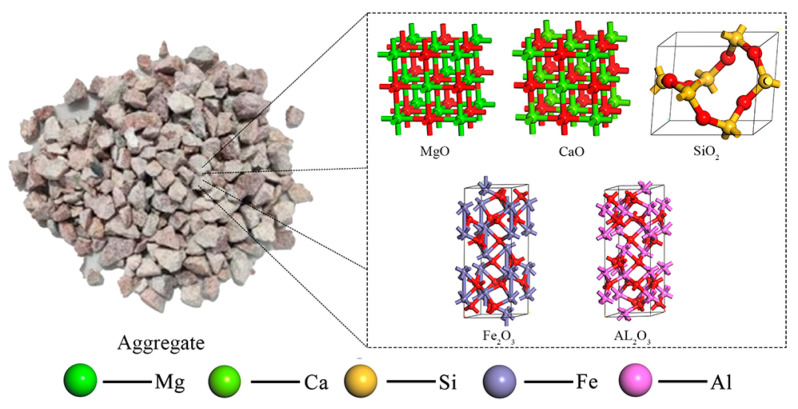
Aggregate molecular model diagram.

**Figure 5:**
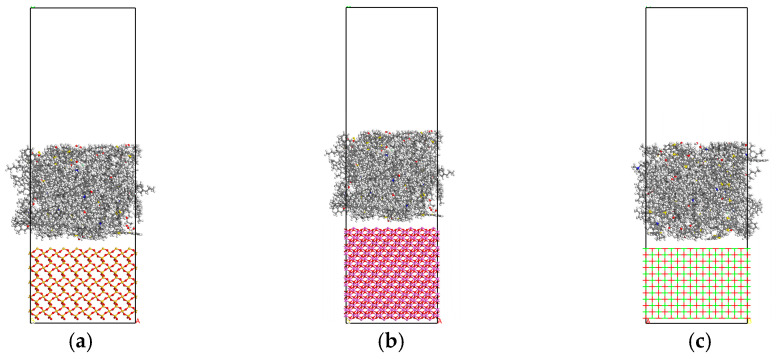
Emulsified asphalt–aggregate interface model diagram: (**a**) emulsified asphalt–SiO_2_ interface model; (**b**) emulsified asphalt–Al_2_O_3_ interface model; (**c**) emulsified asphalt–CaO interface model.

The mineral aggregates such as sand, granite, and limestone commonly used in actual production are a mixture with a complex chemical composition. The main chemical components are SiO_2_, CaO, Al_2_O_3_, Fe_2_O_3_, MgO, etc., as shown in Figure 4. Since the molecular dynamics are difficult to be used to construct a mixed model of various chemical components, this paper uses a single-component model to characterize the mineral aggregates during modeling. CaO, SiO_2_, and Al_2_O_3_, having a high content in the common mineral aggregates, are selected to represent alkaline, acidic, and neutral oxides, respectively. The mineral aggregate model is established, and its lattice parameters are shown in Table 5. Firstly, the single-crystal structure of CaO, SiO_2_, and, Al_2_O_3_ is cut in the direction of [0 0 1] [3]. To make the simulation results closer to the actual value, it is necessary to establish the supercell structure. In this paper, an 8 × 8 supercell structure is constructed. By combining the emulsified asphalt limited in the Z-axis direction with the oxide aggregate, the emulsified asphalt–aggregate interface model is obtained, as shown in Figure 5.

### 3.4. Simulation Method

In the simulation process, periodic boundary conditions were used to relax the emulsified asphalt and emulsified asphalt–aggregate interface model. The truncation radius was set to 12.5 Å, and the temperature was set to 298 K. After relaxation, the kinetic simulation was carried out under the conditions of isothermal isobaric ensemble (NPT) and canonical ensemble (NVT). The time step was 1 fs, the relaxation time was 1 ns, and the results were outputted every 5000 steps to collect the data after the model was stable.

## 4. Results and Discussion

### 4.1. Density

Comparing the model with the experimentally measured density is the most commonly used method to verify the validity of the model. The density of the emulsified asphalt model is calculated using a three-dimensional periodic model using the NPT ensemble at one atmospheric pressure. The simulation data are shown in Table 6.

The difference between the simulated density of CTAB emulsified asphalt and the experimental value is very small, so it can be considered that the simulated density of emulsified asphalt at 298 K is consistent with the actual situation.

### 4.2. Cohesive Energy Density and Solubility Parameters of Emulsified Asphalt

Cohesion energy is usually used to measure the magnitude of the interaction force between molecules [11]. The cohesive energy is the total energy per mole of molecules gathered or the total energy consumed to separate them. The calculation formula is shown in Formula (1). The cohesive energy density is the cohesive energy per unit volume, and the calculation formula is shown in Formula (2); the solubility parameter *δ* is usually used as an important index to measure the intermolecular force. Its physical meaning is the square root of the cohesive energy density of the material, and the calculation formula is shown in Formula (3) [12].

(1) $$E_{\mathrm{coh}}=\left[E_{\mathrm{inter}}\right]=\left[E_{\mathrm{total}}\right]-\left[E_{\mathrm{intra}}\right]$$

(2) $$CED=E_{\mathrm{coh}}/V$$

(3) $$\delta =\sqrt{CED}$$

Among them, [*E_inter_*] is the total energy between all the molecules, [*E_tota_*_l_] is the energy of the whole system, [*E_intra_*] is the intramolecular energy, [*E_coh_*] is the cohesive energy of the molecular system, *CED* is the cohesive energy density, and *δ* is the solubility parameter. The simulation results are shown in Table 7, and the solubility parameters and cohesive energy density are within the reference range, indicating that the molecular model of emulsified asphalt established above is reliable and can be used for interface model research.

### 4.3. Analysis of the Bonding Effect of the Emulsified Asphalt–Aggregate Interface

For the interface, the adhesion work can define the reversible work per unit area required to separate the interface between α and β to generate two free surfaces, that is,

(4) $$W_{\alpha \beta }=\left(E_{\alpha }+E_{\beta }-E_{\alpha \beta }\right)/A$$

In the formula, *E_α_*, *E_β_*_—_the energy of the *α* phase and *β* phase after complete relaxation (Kcal/mol), *E_αβ_*—the total energy of the two interface models, *A*—area of the interface (Å^2^).

The research focus of the water damage mechanism of emulsified asphalt is to analyze the change of adhesion performance, that is, to analyze the change of adhesion performance between emulsified asphalt molecules and mineral aggregates with different water contents. The lammps software was used to calculate the adhesion work of the established asphalt–aggregate interface model, and the changes of the adhesion work of the interface model with water contents of 0.1%, 0.5%, 1.0%, and 1.5% were compared horizontally.

**Figure 6:**
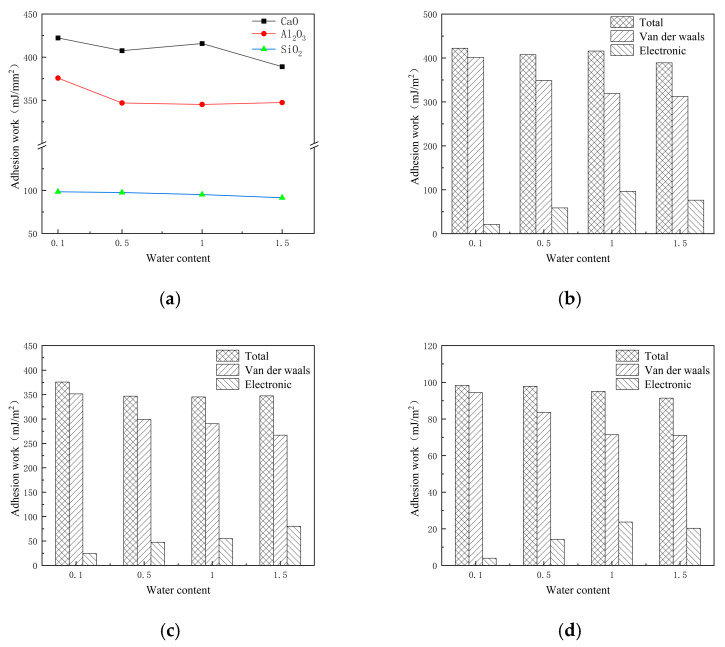
The adhesion work between emulsified asphalt and different oxides under: (**a**) emulsified asphalt–aggregate adhesion work under different water contents; (**b**) adhesion work of emulsified asphalt–CaO under different water contents; (**c**) adhesion work of emulsified asphalt–Al_2_O_3_ under different water contents; (**d**) adhesion work of emulsified asphalt–SiO_2_ under different water contents.

Figure 6a shows the adhesion work between emulsified asphalt and different oxides under different water contents. It can be seen that different oxides have a great influence on the adhesion work. The work of adhesion between the three oxides and asphalt from large to small is CaO > Al_2_O_3_ > SiO_2_, indicating that alkaline oxides are more likely to adsorb asphalt than acidic oxides. Figure 6b,c,d show the adhesion work of emulsified asphalt–aggregate under different water contents. It can be seen that the van der Waals force plays a major role in the adhesion work of the asphalt and oxide interface, and electrostatic force plays a secondary role in the adhesion work of the asphalt and oxide interface. The adhesion work of asphalt and the oxide is the largest when the water content is 0.1%. At this time, the adhesion strength of asphalt and the oxide is the largest. Then, the adhesion work of asphalt and the oxide decreases with the increase in water content, indicating that the increase in water content will reduce the adhesion strength of the interface.

**Figure 7:**
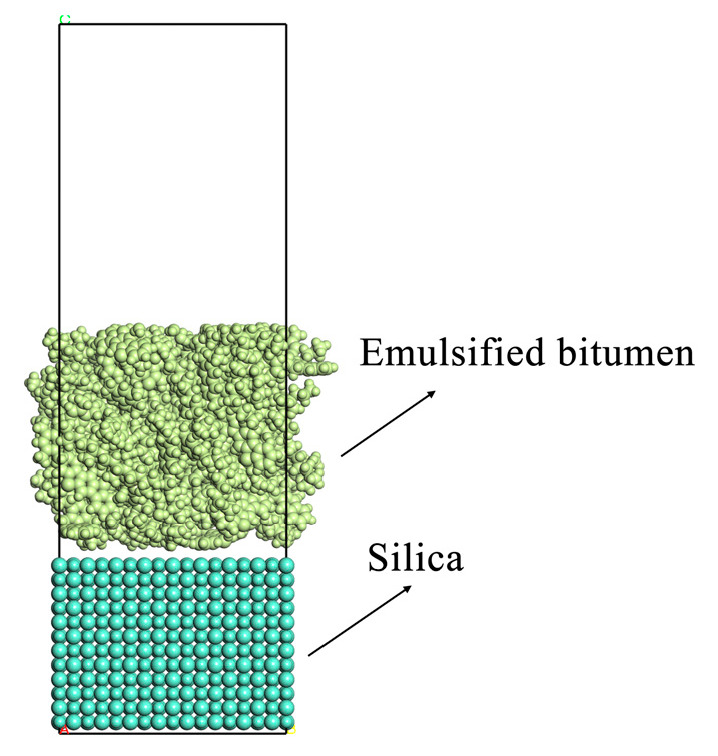
Emulsified asphalt–aggregate tensile diagram.

The tensile failure and cohesive strength of asphalt binder can be determined via a direct tensile test in traditional laboratory tests. In this study, MD was used to simulate the direct tensile test to study the phenomenon of adhesion and cohesion failure of asphalt binders at the atomic scale under the influence of water molecules. As shown in Figure 7, a two-layer system consisting of emulsified asphalt and aggregate is constructed. During the tensile simulation, the aggregate at the bottom is fixed, and the emulsified asphalt layer moves upward at a constant speed of 10 Å/ps. The fixed part of the aggregate makes the whole system not move upward together.

The form of interface rupture is determined by comparing the adhesion energy of different interfaces and the cohesive energy of asphalt during the pull-up process. When the interfacial energy of asphalt and the aggregate is less than the interfacial energy between asphalt, it can be considered that micro-cracks occur at the bond between asphalt and the aggregate, and adhesion failure occurs. When the interfacial energy of asphalt and the aggregate is less than the interfacial energy between asphalt, micro-cracks first appear inside the asphalt, the asphalt is pulled off, and the whole cohesive failure occurs.

**Figure 8:**
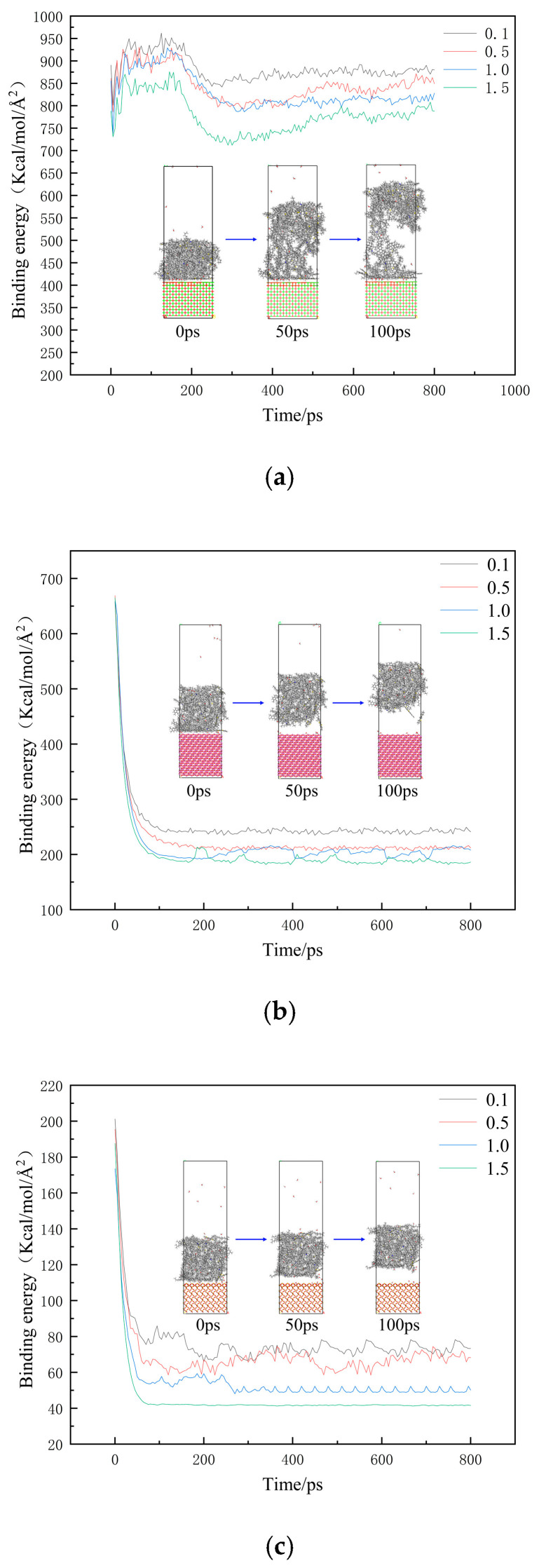
Change diagram of tensile binding energy of emulsified asphalt-aggregate interface with different water content: (**a**) The change diagram of tensile binding energy of emulsified asphalt-CaO interface with different water content; (**b**) Different water content of emulsified asphalt-Al_2_O_3_ interface tensile binding energy change diagram; (**c**) Different water content of emulsified asphalt-SiO_2_ interface tensile binding energy change diagram.

From Figure 8, it can be seen that with the increase in water content, the binding energy between emulsified asphalt and the aggregate decreases continuously, and during the drawing process, the binding energy between the emulsified asphalt and CaO fluctuates within a small range, and the binding energy between the emulsified asphalt and SiO_2_ and Al_2_O_3_ decreases. The rate is positively correlated with the amount of water content. This is because as the degree of water damage increases, the adhesion effect between the emulsified asphalt and SiO_2_ and Al_2_O_3_ gradually decreases under the action of water molecules. After molecular dynamics simulation, the originally free water molecules have been adsorbed on the aggregate surface. In addition, it can be seen from Figure 8a that the damage of CaO and the emulsified asphalt system first appears inside the emulsified asphalt during the stretching process. Under the action of tension, the distance between emulsified asphalt molecules gradually increases. At 50 ps, the distance between emulsified asphalt molecules has been produced. Obviously, the distance is completely broken at 100 ps, which can be considered as the distance between molecules gradually increases and the molecular interaction is reduced. The damage first appears inside the emulsified asphalt, and the system is cohesively damaged; Figure 8b,c show that the binding energy of Al_2_O_3_ and SiO_2_ with the emulsified asphalt first undergoes a rapid decline stage and then enters a stable stage. At 50 ps, the asphalt and mineral aggregate have been completely separated. At this time, the attraction of the molecules between the interfaces is very small, and the system has adhesion damage. Since the molecular dynamics simulation is at the nanometer level, the micro-cracks inside the asphalt mixture begin to appear macroscopically.

### 4.4. Axial Concentration Distribution of Water Molecules

The research goal of this section is the diffusion behavior of water molecules on the surface of the emulsified asphalt–aggregate. The state of water molecule aggregation will be used as an indirect basis for the analysis of water damage in an emulsified asphalt mixture. The diffusion model of different oxide aggregates is established to study the role of water molecules in the whole process of movement and adsorption to aggregates. The mechanism of water damage is explained from the molecular point of view, and the influencing factors of diffusion behavior (aggregate type) are analyzed.

**Figure 9:**
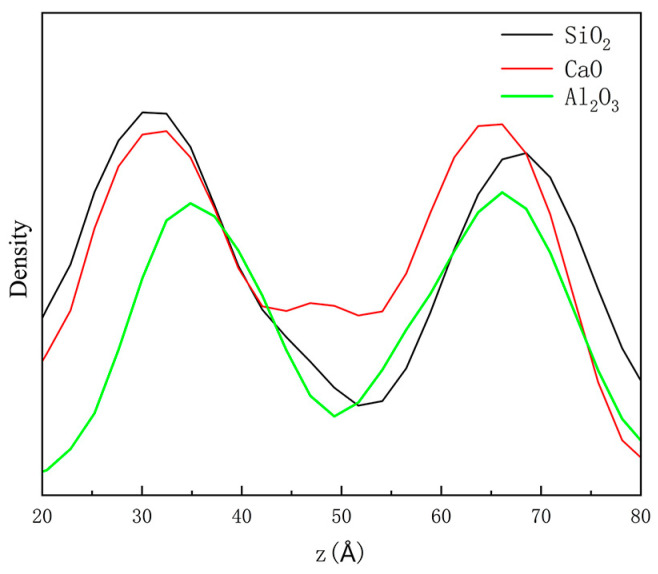
Distribution of axial concentration of water molecules.

The axial concentration distribution of water molecules can well reflect the distribution of water molecules in the *Z*-axis direction (perpendicular to the direction of the crystal substrate) in the emulsified asphalt–aggregate model, to explore the adsorption relationship of water molecules at different crystal interfaces. As shown in Figure 9, the aggregation degree of the density distribution of water molecules in the emulsified asphalt at the crystal interface is higher than that in the vicinity, and there is an obvious peak at the emulsified asphalt–oxide interface, indicating that the water molecules in the emulsified asphalt will gradually diffuse to the crystal interface, eventually causing emulsified asphalt water damage.

## 5. Conclusions

Based on the method of molecular dynamics, this paper uses the lammps software to establish a four-component cetyltrimethylammonium bromide emulsified asphalt, summarizes and analyzes the microscopic factors of water damage of an emulsified asphalt cold recycled mixture, and draws the following conclusions through the analysis of the simulation results:

(1) The van der Waals force plays a major role in the adhesion performance of the emulsified asphalt mixture, and electrostatic force plays a secondary role. Water molecules have a great influence on the electrostatic force energy, and the electrostatic force energy increases with the increase in water content of the emulsified asphalt.
(2) The different chemical composition of aggregate has a great influence on the adhesion work between the emulsified asphalt and aggregate interface. The adhesion work between the three oxides and emulsified asphalt from large to small is CaO > Al_2_O_3_ > SiO_2_. The adhesion strength between the alkaline oxide and emulsified asphalt is higher than that between the acid oxide and asphalt. Under the action of external force, the specific performance is as follows: the failure mode of an alkaline oxide-emulsified asphalt system is a cohesive failure, and the failure mode of an acidic and neutral oxide-emulsified asphalt system is an adhesive failure.
(3) The main factor in emulsified asphalt–aggregate water damage is the enrichment of water molecules at the interface. The water molecules in the emulsified asphalt migrate and aggregate to the interface, and compete with asphalt molecules for the adsorption sites on the oxide surface, resulting in a decrease in the bonding performance of the emulsified asphalt–aggregate interface.

## Figures and Tables

**Table 1:** Main performance indexes of matrix asphalt.

Items	Technical Requirement	Result
Needle penetration (25 °C)/0.1 mm	60~80	69.2
Softening point/°C	≥45	47.5
Ductility (15 °C)/cm	≥100	169
Density (25° C)/g·cm^−1^	Actual measurement	1.013

**Table 2:** Matrix asphalt content table of each component.

Component	Content (%)
Saturates	11.62
Aromatics	32.51
Resins	37.62
Asphaltenes	18.25

**Table 3:** Main performance indexes of emulsified asphalt.

Test Items		Technical Requirement	Result
Residual on sieve (1.18 mm)		≤0.1%	0.07%
Evaporated residue	Density/g·cm^−1^	Actual measurement	0.986
	Residual fraction Content/%	≥50	54
	Needle penetration (25 °C)/0.1 mm	45~150	72
	Ductility (15 °C)/cm	≥40	76

**Table 4:** The molecular mix ratio of emulsified asphalt under different soaking water

Component	Molecular Signature	Molecular Formula	Number of Molecules	Water Mass Fraction%			
				0.1%	0.5%	1%	1.5%
Saturates	SA-A	C_30_H_62_	4	4.96277	5.01312	4.98649	4.96277
	SA-B	C_35_H_62_	4	5.66764	5.72514	5.69474	5.66764
Aromatics	AR-A	C_35_H_44_	11	15.00038	15.15257	15.07209	15.00038
	AR-B	C_30_H_46_	13	15.51379	15.67118	15.58795	15.51379
Resins	RE-A	C_40_H_59_N	4	6.50143	6.56739	6.5325	6.50143
	RE-B	C_40_H_60_S	4	6.72515	6.79338	6.7573	6.72515
	RE-C	C_18_H_10_S_2_	15	12.78167	12.91134	12.84277	12.78167
	RE-D	C_36_H_57_N	4	5.91386	5.97386	5.94213	5.91386
	RE-E	C_29_H_50_O	5	6.08451	6.14624	6.11359	6.08451
Asphaltenes	AS-A	C_42_H_54_O	3	5.0607	5.11204	5.08489	5.0607
	AS-B	C_66_H_81_N	2	5.21353	5.26642	5.23845	5.21353
	AS-C	C_51_H_62_S	3	6.22465	6.2878	6.2544	6.22465
H_2_O		H_2_O		0.10725	0.53398	1.06228	1.53297

**Table 5:** Cell constant of oxides.

Composition Type	The Crystal Lattice Constant					
	Length/Å			Angle/(°)		
	a	b	c	α	β	γ
CaO	4.8105	4.8105	4.8105	90	90	90
SiO_2_	4.913	4.913	5.4052	90	90	120
Al_2_O_3_	4.759	4.759	12.991	90	90	120

**Table 6:** The density of emulsified asphalt with different water contents.

Moisture Content	0.1%	0.5%	1%	1.5%
Density/g·cm^−1^	0.980	0.978	0.978	0.974

**Table 7:** Cohesive energy density and solubility simulation value and reference value parameters of emulsified asphalt with different water contents.

	Value of Simulation				Value of Reference
	0.1	0.5	1	1.5	
*CED* (10^8^ J/m^3^)	3.291	3.467	3.516	3.52	3.31~3.73
*δ*/(10^6^ J/m^3^)^0.5^	18.121	18.619	18.75	18.761	18.19~19.31

## Data Availability

The data are contained within the article.
